# Data on the effect of sub-epithelial connective tissue graft with hydroxyl-apatite for furcation treatment in Indians

**DOI:** 10.6026/97320630018364

**Published:** 2022-04-30

**Authors:** Ena Sharma, Neha Jain, Amit Lakhani, Deepak Sharma, Shivani Dhawan, Ridhima sood

**Affiliations:** 1 Rayatbahra Dental College and Hospital, Mohali India; 2 Bayhi Dental Care San Bruno California, USA; 3 Dr BR Ambedkar State Institute of Medical Sciences, SAS Nagar Punjab, India; 4 H.P. Government Dental College & Hospital Shimla, Himachal Pradesh, India; 5 Maharishi Markandeshwar College of Dental Sciences and Research, Mullana, Haryana, India

**Keywords:** Grade II furcation defects, sub-epithelial connective tissue grafts, Bio-OssTM bovine bone graft, guided tissue regeneration

## Abstract

Grade II furcation defect has a great potential for regeneration. Attempts have been made to regenerate lost periodontium in furcation defects through variety of approaches. A total of 22 patients with Grade II furcation defect were examined. The patients were sequentially grouped into three groups namely, (Group A) subepithelial connective tissue graft as a barrier membrane, (Group B) bovine bone graft material Bio-OssTM and (Group C) with the combination of the two. The treatment modalities show that there was a strong improvement in the resolution of the grade II furcation defects. The reduction in horizontal furcation defect measurement was 20.63% for group A. The change was found to be moderate (p=0.023*). The % change is 20.97% for group B and it is statistically significance (p = 0.001**). The % change was 20.78% in Group C with a good statistical significance (p=0.003**). Complete obliteration of the defect clinically and radio-graphically was observed for grade II furcation defect. Thus, the effect of sub-epithelial connective tissue graft with hydroxyl-apatite for furcation treatment in Indians is documented in this report.

## Background:

Furcation defects represent a formidable problem in the treatment of periodontal disease related to the complex and irregular anatomy of dental furcation. Moreover, the response to therapy is complicated by the presence of a greater radicular surface offered to bacterial toxins, calculus build up, the distal location in the arch and the difficult access. This may conceivably impair both self-performed and professional plaque control procedures in the furcation area limiting their effective management. [1] Furcation defect is a lesion with the presence of two osseous walls (inter-radicular septi), an intra osseous defect with the greatest potential for regeneration. Nonetheless, attempts have been made to regenerate lost periodontium in furcation defects through a variety of approaches. Techniques for clinical usage include bone replacement grafts, guided tissue regeneration via barrier membranes and combinations of regenerative techniques including adjunctive measures such as root conditioning and/or coronal flap positioning. [2] The use of sub epithelial connective tissue graft offers a combination of both the pedicle flap and free gingival graft. [3,4] The pedicle flap retains its apical blood supply and therefore survives over a vascular root surface. The free gingival graft supplies are a type of connective tissue which ensures thickness and keratinization [5].

The ability to combine both procedures offer the flexibility not achieved by either technique alone [6]. The clinically successful regeneration at the furcation sites is determined by the elimination or reduction of the horizontal and vertical components of the lesion. Clinical trials using various membranes for the regenerative treatment of grade II furcation defects have not shown significant bone formation. However, when combining porous hydroxyl-apatite with a poly tetra fluoro-ethylene membrane in the treatment of grade II furcation have shown significant gain in both vertical and horizontal bone levels. [3] Porous bone minerals (Bio-OssTM, Osteo Health) are xeno-graphic bone graft materials and are obtained by extracting proteins from bovine cancellous or cortical bone. These bone minerals are biocompatible, non-antigenic and permits physiologic vascular ingrowths. This also permits complete integration and incorporation in to bone and possess the similar structure as that of bone. Auto-genous, allograft and artificial bone graft materials are used for space maintenance in GTR. However, in the combined use of the GTR method and bone graft materials selectively depopulate periodontal ligament cells on the previously periodontitis-affected root surface. [4] Data with sub-epithelial connective tissue graft including the periosteum as a barrier membrane and resorbable Hydroxyapatite bone substitutes, in terms of bone-fill in furcation defects individually is known. Therefore, it is of interest to analyze subepithelial connective tissue graft including the periosteum as a barrier membrane, resorbable Hydroxyapatite bovine bone graft material individually and in combination in the treatment of grade II furcation defects.

## Materials & Method:

Subjects with grade II furcation involvement/defects were recruited among the patients based on Inclusion criteria of systemically healthy subjects aged between 20 and 55years, presence of grade II molar furcation defects, persistent vertical probing pocket depth of≥5 mm following phase I therapy and presence of proximal bone coronal to the furcation defect.

## Study design:

All the patients underwent phase I therapy prior to the surgical procedure. Routine blood investigations were carried out along with IOPA radiograph for the region affected with inter-radicular bone loss to assess the level of inters dental bone. Alginate impressions were made for the upper and lower arch for the preparation of a study model. All patients were re-evaluated and baseline measurements were recorded after phase I. Parameters were recorded at baseline and 6 months interval.

## Baseline measurements:

Probing Pocket Depth (PPD), Clinical Attachment Level (CAL) and Gingival Recession (GR) was measured with a William's periodontal probe. A colour-coded calibrated Nabers' probe was used to assess the presence of grade II furcation defects clinically. A custom acrylic stent for positioning of the Williams’ periodontal probe for vertical measurement and the Nabers' probe for the horizontal measurement of the furcation defect was prepared using a clear self-cure acrylic resin.

## Radio graphic assessment:

A standardized intra-oral periapical radiograph was taken. Radiographs were made at base line and 6 months post-operatively. The presence of radiographic evidence of the grade II furcation defect and the extent of obliteration of the defect were visualized at base line and 6 months post-operatively. Measurement of the distance from the apical most point of contact area to the alveolar bone crest in the inter-radicular region on the intraoral periapical radiograph using are amer with a rubber stopper, was measured with a digital Vernier Caliper and rounded to the nearest 0.1 mm.

## Surgical procedure:

### Pre-surgical preparation:

 Pre-surgical rinse (intra-oral asepsis) was performed with 0.2% chlorhexidene gluconate mouth wash.

### Recipient site preparation:

Buccal and lingual sulcular incision were made, using a Swann-Morton no.15 blade on a BP handle following administration of Local anesthesia of 2% Lignocaine chloride containing adrenaline at a concentration of 1:2,00,000. This is to split the inter-dental papilla to facilitate periodontal flap reflection with maximum care taken to preserve as much inter proximal tissue. Full-thickness muco-periosteal flaps were raised using a periosteal elevator. Meticulous defect Debridement, debridement of the surgical site and root planing were carried out using curettes (Gracey, Hu-Friedy, and Chicago, IL,USA) and a piezoelectric ultrasonic scaler (Piezon, Biosonic TM).

### Donor site (harvesting of the graft):

Periosteal grafts were harvested from the palatal mucosa of the same side using a "trapdoor" technique following greater palatine nerve block. At the palatal donor site, horizontal incision, made with a #15 no. blade ∼3 mm from the free gingival margin. A second incision was made with a #15 no. blade under mining and splitting the palatal flap. Vertical incisions were placed anteriorly and posteriorly beneath the palatal flap, and another horizontal incision was made apically on the connective tissue to sever the graft tissue. The underlying connective tissue including periosteum was removed using a sharp periosteal elevator. The donor site was sutured using direct loop interrupted suturing technique, with 4-0 braided silk suture material.

### Soft tissue graft preparation:

The soft tissue graft was trimmed and adapted over the furcation defect into the envelope type recipient flap, planned to facilitate maximum coverage, with the perio steal side facing the defect, stabilized using 5-0 cromic vicryl resorbable suture material using horizontal mattress sling suturing technique. Group A sites, received subepithelial connective tissue graft along with the periosteum as obtained and adapted over the defect in the aforementioned manner. The periodontal flaps were repositioned and sutured using 3-0 braided silk suture material, direct interrupted loop suturing technique, ensuring complete coverage of the graft material. Group B sites, received only resorbable HA bovine bone graft material, had the surgical site prepared in the similar manner as mentioned above. The required amount of bone graft material (BIO-OSS) TM was wetted with normal saline and placed on the prepared site of the defect area, closed by flap replacement and sutured. Group C sites, received both the bone graft material, resorbable HA bovine bone graft material, following the surgical site preparation in the similar manner as stated earlier. The required amount of bone graft material (BIO-OSS) TM was wetted with normal saline and placed on the prepared site of the defect area, as described previously for group B, as well as the sub epithelial connective tissue graft along with the periosteum, as mentioned for group A, also treated in the aforementioned manner of graft placement, flap repositioning and suturing. All sites were covered with period on taldressing (CoePack).Post-operative instructions were given.

### Post-operative care:

Post operative instructions were provided to each patient verbally. To minimize potential infection patients were prescribed antibiotics such as amoxicillin, 500 mgTID for 5 days or azithromycin, 500 mg OD for 3 days (in case of penicillin allergy) and analgesics, such as, ibuprofen 400 mg TID for 3 days. Periodontal dressing and sutures were removed 2 weeks post-operatively following irrigation with normal saline and povidone iodine.

## Results and Discussion:

A total of 22 patients, 7 female and 15 male patients aged between 20 - 50 years, with 31 grade II furcation defects (maxillary, buccal or mandibular, buccal &/or lingual) requiring periodontal flap surgical, regenerative procedure, participated in the study after the fulfillment of the inclusion and exclusion criteria. The amount of reduction in probing depth within the three groups was statistically highly significant (p=0.002**for group A; p<0.001**for group B and p<0.001** for group C). However, no statistical significant difference in the probing depth reduction was found when the results were compared among the three groups (Table 1 and Figure 1 - see PDF). The % change in CAL groups A, B and C were 41.67%, 34.99% and 37.66% respectively. All these reductions within the groups were strongly statistically significant (p=0.005**, p<0.001**and=0.005**for groups A, B and C, in that order) but no difference were seen when the results were compared among the three groups (Table 1 & Figure 2 - see PDF).

## Furcation defect measurements:

The relative horizontal measurements of the furcation defects, made using an acrylic stent, with a mean of 15.947 mm ± 3.196 pre operatively, which was reduced to a mean of 12.170 mm ± 1.752 (% change = 20.63%) for group A. The change was found to moderately statistically significant (p=0.023*). For group B, the mean at base line was16.411 mm ± 1.973 which reduced 6 months post operatively to 12.964 mm ± 2.543,(% change = 20.97%) which is of strong statistical significance (p=0.001**). In group C the mean horizontal measurement preoperatively was 16.540 mm ± 3.322 whichreducedtoameanof12.91 mm ± 2.661(%change=20.78%) with a strong statistical significance (p=0.003**). No difference was seen when the results were compared among the three groups (Table 2 and Figure 3 - see PDF).

## Vertical measurement:

The relative vertical measurement obtained using the custom acrylic stent, calculated mean of 13.235 mm ± 2.363 at base line for group A which was reduced to 11.237 mm ± 1.063 at 6 months follow up check up (% change = 14.36%) and was found to be of moderate statistical significance (p=0.036*). For group B the mean value preoperatively was 12.430 mm ± 1.950 which was found to be reduced to 10.546 mm ±1.842 (% change = 15.15%) which was found to be strongly statistically significant (p<0.001**). For group C, the mean at baseline of 14.180 mm ±2.939 was reduced.686 mm ±2.445 (%change = 18.30%) which was also strongly statistically significant (p < 0.001**). No difference were seen when the results were compared among the three groups (Table 2 and Figure 4 - see PDF).

## Radiographic measurement Vertical measurement of the furcation defect:

The % of the radiographic measurement changes for group A was 40.93%); for group B (% change = 47.62%) and for group C was % change = 45.5%). This was found to be of no statistical significance (p=0.055+) when the results were compared among the three groups (Table 3 and Figure 5 - see PDF). Patients with 31 grade II furcation defects participated in this study satisfying the inclusion and exclusion criteria. There were 2 cases with grade II cervical enamel projections that required osteoplasty as its presence would otherwise retard healing in the furcation region and hinder any chances of regeneration.

There were no incidences of post operative complications immediately following surgery or on re call visits. All treated patient maintained excellent oral hygiene throughout the observation period of 6 months. The results of this study showed that all the three groups showed moderately statistically significant improvement. The clinical effects of bio absorbable regenerative material in comparison with an ePTFE non-resorbable membrane in the treatment of mandibular buccal class II furcation defects, the results suggested that 12 months after surgery, similar clinical improvements can be obtained in GTR therapy of buccal class II furcation lesions, whether bio absorbable PGA/PLA membranes or non-resorbable PTFE are used. The development of SCTG has significantly improved treatment options and predictability. It should be noted that the connective tissue graft was used to increase the width of keratinized gingival for several years.

The use of subepithelial connective tissue graft offers a combination of both the pedicle flap and free gingival graft. The pedicle flap retains its apical blood supply and therefore survives over an avascular root surface. The free gingival graft supplies are silient type of connective tissue with a genetic predisposition which ensures thickness and keratinization. The ability to combine both procedures offers the flexibility to achieve by either technique alone.39. The periosteum has been shown to have the potential to stimulate bone formation when used as a graft material in human and animal studies. Case reports of the use of periosteum in intra bony defects have shown evidence of bone fill and improvements in probing pocket depths. Periosteum can easily be harvested, does not require a second surgical procedure for removal, and does not pose any risk of disease transmission to the patient. In this study, for group A, the mean probing pocket depth at baseline reduced from 5.600 mm ± 1.645 to 3.000 mm ± 0.534 6 months postoperatively (%change = 49.11%) which was statistically highly significant (p=0.002**). The mean clinical attachment level in group A 6.000 mm ± 1.699 preoperatively was reduced to a mean of 3.750 mm ± 1. 488 postoperatively which was strongly statistically significant (p=0.005**). The relative horizontal measurements of the furcation defects, made using an acrylic stent, with a mean of 15.947 mm ± 3.196 preoperatively, which was reduced to a mean of 12.170 mm ± 1.752 (% change = 20.63%) for group A. The change was found to moderately statistically significant (p=0.023*). The relative vertical Measurement obtained using the custom acrylic stent, calculated a mean of13.235 mm ± 2.363 at baseline for group A which was reduced to 11.237 mm ± 1.063 at 6 months follow up check up (% change = 14.36%) and was found to be of moderate statistical significance (p=0.036*). The mean of the radiographic measurement at baseline for group A was 1.588 mm ± 0.687 which reduced to 1.02 mm ± 0.792 (% change =40.93%)which was found to be of only moderate statistical significance (p=0.019*). The results thus obtained are in accordance with the studies as conducted by Lekovic et al. [7,8]. A number of studies have demonstrated the ability of bovine derived xeno graft (BDX) Auto genous, allograft, and artificial bone graft materials are used for space maintenance in GTR. However, the combined use of the GTR method and bone graft materials selectively depopulated periodontal ligament cells on the previously periodontitis-affected root surface [9-13].

The available bovine derived HA like Bio-Oss TM has been reported to have good tissue acceptance with natural osteo-trophic properties. In this study, in the group B patients, the mean probing pocket dep that base line was5.181 mm ± 0.603 which reduced to 2.636 mm ± 0.674 at the end of the study period and % change was 49.12% which was statistically highly significant (p<0.001**).The mean clinical attachment level in group B was mean of 5.455 mm ± 0.687 mm preoperatively and mean of 3.545 mm ± 1.634 postoperatively, strongly statistically significant (p<0.001**). The relative horizontal measurements of the furcation defects, made using anacrylic stent, the mean at baseline was 16.411 mm ±1.973 which reduced 6 months post operatively to 12.964 mm ± 2.543, (% change = 20.97%) which is of strong statistical significance (p=0.001**). The relative vertical measurement obtained using the custom acrylic stent, calculated a mean preoperatively of 12.430 mm ± 1.950 which was found to be reduced to 10.546 mm ±1.842 (%change=15.15%) which was found to be strongly statistically significant (p<0.001**). The mean of the radiographic measurement at baseline for group B at baseline was 1.617 mm ± 0.592 which was found to have reduced to 0.847 mm ±0.878 (%change=47.62%) which was also found to be moderately statistically significant (p=0.010*). The results with respect to group B were found to be in accordance with data shown elsewhere [14]. In this study, for group C the mean probing pocket depth at baseline was 5200 mm ±1.03 which reduced to 2.555 mm ± 0.527 at 6 months postoperatively with % change of 51.28%. The amount of reduction in probing depth within the three groups was statistically highly significant (p<0.001**). Mean clinical attachment level reduced from mean of 5.900 mm ±1.595 preoperatively to mean of 3.777 mm ±1.301postoperatively, strongly statistically significant (p=0.005**). Mean horizontal measurement pre operatively was 16.540 mm ± 3.322 which reduced to a mean of 12.911 mm ±2.661 (%change=20.78%) with a strong statistical significance (p=0.003**). The relative vertical measurement obtained using the custom acrylic stent the mean at base line of 14.180 mm ± 2.939 was reduced to 11.686 mm ± 2.445 (%change = 18.30%) which was also strongly statistically significant (p<0.001**). The mean of the radiographic measurement at baseline for group C reduced from 1.618 mm ± 0.853 to 6 months post operative mean value of 0.970 mm ± 0.806 (% change =45.5%), was found to be of no statistical significance (p=0.055+). The results of the present study with respect to group C are in accordance with the results as obtained in previous studies conducted by Camelo et al. [15], Yamada et al. [16] as well as Belal et al. [17]. No difference was seen when the results of the various parameters were compared among the three groups. The present study has shown that the three methods are effective in the treatment of grade II furcation defects in terms of reduction in probing pocket depth, gain in clinical attachment level, reduction in relative horizontal and vertical furcation defect measurements and vertical radiographic furcation defect measurement that has strong statistical significance while none of the methods proved to be superior statistically on comparison. Complete obliteration of the defect clinically and radio graphically was observed for 7grade II furcation defects out of which 4 belonged to group B (Bio-OssTM bovine bone graft), 2 belonged to group C (bone graft and SCTG) and1, to group A (SCTG). Therefore, the selection of the method for the treatment of grade II furcation defects from among the three methods used in the study, in an attempt at regeneration in there into the defect, would be governed by the characteristic features of the defect. Data shows that all the three methods are effective in the treatment to grade II furcation defects while no single method was superior to the other.
